# Prostate-Specific Membrane Antigen Positron Emission Tomography (PSMA-PET) in Initial Staging of Prostate Cancer Patients: The Beginning of a New Era

**DOI:** 10.3390/medicina61050924

**Published:** 2025-05-20

**Authors:** Juan Gómez Rivas, Irene de la Parra, Sarelis Infante, Laura Ibañez, Beatriz Gutierrez Hidalgo, María Nieves Cabrera, Javier Puente, Noelia Sanmamed, Luis Enrique Ortega Polledo, María Isabel Galante, Jesús Moreno Sierra

**Affiliations:** 1 Department of Urology, Clínico San Carlos University Hospital, Institute for Health Research, Universidad Complutense de Madrid, 28040 Madrid, Spain; sarelis91@gmail.com (S.I.); livemir9@gmail.com (L.I.); leortegapolledo@gmail.com (L.E.O.P.); m.isabel.galante@gmail.com (M.I.G.); dr_jmoreno@hotmail.com (J.M.S.); 2 Department of Urology, Ramón y Cajal University Hospital, 28034 Madrid, Spain; irn_parra@hotmail.com; 3 Department of Urology, University Hospital of Canarias, 38320 Tenerife, Spain; bea_gutih@hotmail.com; 4 Department of Nuclear Medicina, Clínico San Carlos University Hospital, Institute for Health Research, Universidad Complutense de Madrid, 28040 Madrid, Spain; marcab06@ucm.es; 5 Department of Medical Oncology, Clínico San Carlos University Hospital, Institute for Health Research, Universidad Complutense de Madrid, 28040 Madrid, Spain; javierpuente.hcsc@gmail.com; 6 Department of Radiation Oncology, Clínico San Carlos University Hospital, Institute for Health Research, Universidad Complutense de Madrid, 28040 Madrid, Spain; noelia_ss@hotmail.com

**Keywords:** prostate cancer, initial staging, PSMA-PET

## Abstract

*Background and Objectives:* Prostate cancer (PCa) is a common disease, with a significant number of patients initially diagnosed with locoregional or distant metastases. This is why it is essential to have imaging tests with sufficient sensitivity and specificity. Given the recognized limitations of traditional imaging methods, PSMA-PET has emerged as a promising tool that may revolutionize the management of PCa. *Material and Methods:* We conducted a comprehensive literature review from August to October 2023 using databases and a review of key clinical guidelines on the topic, focusing on the sensitivity and specificity of PSMA-PET, its use in detecting lymph node metastases (LNm), its integration into nomograms, its comparison with conventional imaging and current guideline recommendations. *Results:* After considering the search strategy, as well as the inclusion and exclusion criteria, four articles and five guidelines were particularly considered in this review. Most of them suggest high specificity and limited sensitivity for ^68^Ga-PSMA-PET, with increased detection rates compared to conventional imaging modalities, especially in high-risk PCa patients. However, it cannot replace an extended pelvic lymph node dissection (ePLND) at this time. *Conclusions:* Although the enhanced sensitivity and specificity of PSMA-PET relative to conventional imaging modalities offers a more precise evaluation of disease extent, prospective studies demonstrating a survival benefit are currently lacking; therefore, caution is advised when making therapeutic decisions.

## 1. Introduction

Prostate cancer (PCa) is the second most common cancer in men, with an incidence of 1.4 million new diagnoses per year and a global mortality of 350.000 people [1]. While most patients are diagnosed with localized tumors, a significant percentage present with locoregional metastases (15%) or distant metastases (5%) at the time of diagnosis, making accurate staging crucial for defining the most appropriate treatment strategy [2,3].

Traditional imaging methods in PCa, such as multiparametric magnetic resonance imaging (mpMRI), contrast-enhanced computed tomography (CT) and technetium-99 m (99mTc)-methylene diphosphonate bone scan (BS), have significant diagnostic limitations [4]. These limitations have encouraged the development of advanced molecular imaging techniques such as prostate-specific membrane antigen positron emission tomography (PSMA-PET), which offers enhanced sensitivity and specificity in PCa imaging [5].

PSMA-PET is an advanced whole-body imaging technique that provides high-contrast visualization of prostate cancer (PCa). PSMA is a glycoprotein that is significantly overexpressed in PCa cells. Radiolabeled small molecules that specifically bind to PSMA enable targeted tumor imaging using PET-CT [6].

Although PSMA-PET has primarily been studied for localized recurrences [7], emerging data support its use in primary staging, particularly for identifying lymph node (LN) enrolment, even in subcentimeter nodes [8,9]. This review synthesizes current evidence on the diagnostic use of PSMA-PET in localized PCa and analyzes the clinical implications of its implementation in patient management.

## 2. Material and Methods

A comprehensive literature review was conducted from August to October 2023 using databases such as PubMed/MEDLINE, ScienceDirect and the Cochrane Library. Keywords included ‘prostate cancer’, ‘positron emission tomography’, ‘prostate-specific membrane antigen (PSMA-PET)’, ‘diagnosis’ and ‘therapy’. Studies related to biochemical recurrence were excluded. Boolean operators “AND”/” OR” were used to combine search terms, and “NOT” was employed to exclude studies focusing on biochemical recurrence. This search strategy was optimized to yield the most relevant studies, particularly from PubMed/MEDLINE.

### 2.1. Study Selection

The selected studies were filtered according to the following inclusion and exclusion criteria:

#### 2.1.1. Inclusion Criteria

- Original research articles, including clinical trials, systematic reviews and research studies.
- Studies involving patients aged 18 years or older.
- Publications from 2008 onwards.
- Articles published in English.

#### 2.1.2. Exclusion Criteria

- Descriptive studies, such as case reports or clinical case series.
- Studies involving pediatric patients (under 18 years old).
- Publications prior to 2008.
- Non-English language publications.

Additionally, a review of key clinical guidelines was conducted, including those from the European Association of Urology (EAU) [10], the European Society for Medical Oncology (ESMO) [11], the American Society of Clinical Oncology (ASCO) [12], the American Urological Association (AUA) [13] and the Advanced Prostate Cancer Consensus Conference (APCCC) [14].

### 2.2. Data Synthesis

Given the limited research on PSMA-PET for primary staging, this review focuses on several key areas: sensitivity and specificity of PSMA-PET, its use in detecting lymph node metastases, its integration into established nomograms, its comparison with conventional imaging and current guideline recommendations.

The studies analyzed are summarized in Table 1. Current guideline evidence is summarized in Table 2.

## 3. Results

After considering the search strategy, as well as the inclusion and exclusion criteria, four articles and five guidelines were particularly considered in this review. The analysis is organized into the following categories:

### 3.1. Sensitivity and Specificity of PSMA-PET

Conventional imaging for PCa staging, such as CT or mpMRI, has low sensitivity for detecting lymph node involvement, with rates of less than 40%. CT and MRI imaging rely on morphological features, with LNs larger than 8 to 10 mm considered suspicious. However, more than 80% of PCa LNMs are smaller than 8 mm [15]. Nowadays, bilateral extended pelvic lymph node dissection (ePLND) during radical prostatectomy (RP) is considered the most precise method for diagnosing LNs involvement [8]; however, it is an invasive diagnostic tool associated with complications, such as lymphocele (3% to 17% of cases) and lower extremity edema in 3% of cases [16]. On these grounds, PSMA PET has been investigated for nodal staging evaluation in intermediate and high-risk PCa.

In 2016, Maurer et al. [8] were the first to publish their results comparing ^68^Ga-PSMA-PET with CT and mpMRI in 130 patients with intermediate- to high-risk PCa scheduled for RP and ePLND. In a patient-based analysis, the sensitivity, specificity and accuracy of ^68^Ga-PSMA-PET were 65.9%, 98.9% and 88.5%, while those of morphological imaging were 43.9%, 85.4% and 72.3%, respectively. In a template-based analysis, the sensitivity, specificity and accuracy of ^68^Ga-PSMA-PET were 68.3%, 99.1% and 95.2%, while those of morphological imaging were 27.3%, 97.1% and 87.6%, respectively.

Subsequently, in 2020, Van Kalmthout et al. [16] compared ^68^Ga PetPSMA and bilateral ePLND during RP. They showed a patient-based sensitivity of 41.5% (95% CI 26.7–57.8) for detecting lymph node metastasis, a patient-based specificity rate of 90.9% (95% CI 79.3–96.6) and positive and negative predictive values of 77.3% (95% CI 54.2–91.3) and 67.6% (95% CI 55.6–77.7). As a result, PSMA-PET led to a change in treatment in 13 patients (12.6%).

### 3.2. Integration of PSMA-PET into Nomograms

The decision to perform an ePLND relies on established preoperative nomograms, including the Briganti 2017 nomogram [17] and the Memorial Sloan Kettering Cancer Center (MSKCC) nomogram [18]. Both tools utilize comparable clinical, biochemical and pathological preoperative variables. In contrast, the Briganti 2019 nomogram [19] enhances this approach by integrating imaging results and histological data from targeted biopsies following multiparametric MRI (mpMRI). The cutoff percentage above which an ePLND is recommended differs across sources: 2% in the National Comprehensive Cancer Network guidelines [20], 5% in the European Association of Urology guidelines [10] and 7% in the Briganti 2019 nomogram [19].

Due to the accuracy of the information provided by PSMA-PET, its inclusion in these nomograms has been considered. A multicenter study conducted in 2019 showed that the addition of PSMA-PET to previously developed nomograms resulted in substantially improved performance in predicting the outcome of ePLND correctly. In terms of AUCs, the AUCs for the Briganti 2017, MSKCC and Briganti 2019 nomograms were 0.70 (95% confidence interval [95% CI]: 0.64–0.77), 0.71 (95% CI: 0.65–0.77) and 0.76 (95% CI: 0.71–0.82), respectively. In addition, after the use of the PET-PSMA, the AUCs were augmented to 0.76 (95% CI:0.70–0.82), 0.77 (95% CI: 0.72–0.83) and 0.82 (95% CI: 0.76–0.87), respectively [21].

### 3.3. Guideline Recommendations

As it is shown, the EAU guidelines [10] reflect that PET-PSMA increases detection rates in PCa patients, especially in high-risk cases, compared to conventional imaging. However, it is unclear whether patients with metastases only detectable with PSMA-PET should be managed using systemic therapies only, or whether they should be treated with aggressive local therapies. The latest EAU guidelines (2024) advocate for the use of PSMA-PET in the primary staging of high-risk prostate cancer due to its superior detection rates compared to conventional imaging, with a strong rating. However, the guidelines also indicate the need for caution, given that the prognostic implications of detecting metastases solely through PSMA-PET remain uncertain. Therefore, further prospective studies are needed to clarify its impact on clinical outcomes [10]. It is worth mentioning that they recommend it with limited supporting evidence in patients with intermediate-risk PCa.

The ESMO guidelines [11] suggest that PET-PSMA has better sensitivity and specificity than CT or BS. Nevertheless, PET-PSMA has not been shown to improve clinical outcomes.

The ASCO guidelines [12] recommend PET-PSMA when conventional imaging modalities are negative o equivocal in high- or very high-risk PCa due to its high sensitivity. However, this high sensitivity of PET-PSMA for detecting low-burden disease may lead to incorrect patient management.

The AUA guidelines [13] state that PET-PSMA is recommended only for high-risk PCa patients, even though currently PET-PSMA it not indicated in the initial stages of PCA.

APCCC [14] recommend using PET-PSMA in cases of high-risk localized PCa, even without having previously used conventional imaging modalities. On other hand, PET-PSMA is not recommended in favorable intermediate-risk disease, and its use in unfavorable intermediate-risk PCa is controversial.

**Table 1:** Most relevant previously published manuscripts on the usefulness of PET-PSMA in localized prostate cancer.

Author	Study Design	Objective	Participants	Results
Hofman MS et al. (2020) [6]	Prospective multicentre study	To evaluate accuracy of first-line imaging (CT or BS versus PSMA-PET) for identifying either pelvic nodal or distant-metastatic disease.	A total of 302 men (with biopsy-proven prostate cancer and high-risk features at ten hospitals in Australia) were randomly assigned. A total of 152 (50%) men were randomly assigned to conventional imaging and 150 (50%) to PSMA PET-CT.	PSMA-PET had a 27% (95% CI 23–31) greater accuracy than that of conventional imaging (92% (88–95) vs. 65% (60–69); *p* < 0·0001). They found a lower sensitivity (38% (24–52) vs. 85% (74–96)) and specificity (91% (85–97) vs. 98% (95–100)) for conventional imaging compared with PSMA-PET.
Maurer T et al. (2016) [8].	Retrospective analysis	To evaluate the diagnostic value of ^68^Ga-PSMA-PET in comparison to morphological imaging (CT and mpMRI) for LN staging in patients with intermediate- to high-risk PCa undergoing RP with ePLND.	130 patients with intermediate to high risk PCa who underwent ^68^Ga-PSMA-PET and subsequent RP.	^68^Ga-PSMA ligands have the potential to replace currently used tracers for PET not only for recurrent PCa but also for primary LN staging.
Van Kalmthout et al. (2020) [16].	Prospective study	Evaluates the diagnostic accuracy of ^68^Ga-PSMA-PET/CT to guide its implementation into clinical practice.	Patients newly diagnosed with PCa who have more than 10% risk for LNMs according to the MSKCC criteria and were considered candidates for ePLND	High specificity and moderate sensitivity for ^68^Ga-PSMA-PET/CT to detect LNM in the initial staging of patients with PCa, negative bone scans and a greater than 10% chance of LNM.
Meijer D et al. (2021) [21].	Multicenter study. Retrospective study.	To determine the predictive performance of the Briganti 2017, MSKCC and Briganti 2019 nomograms with the addition of PSMA-PET.	All 757 eligible patients who underwent a PSMA-PET prior to RARP and ePLND.	The addition of PSMA-PET to the previously developed nomograms showed substantially improved predictive performance.

^68^Ga-PSMA-PET: prostate-specific membrane antigen positron emission tomography with Gallium 68. CT: computed tomography. MpMRI: multiparametric magnetic resonance imaging. RP: radical prostatectomy. EPLND: extended pelvic lymph node dissection. LN: lymph node. LNM: lymph node metastases. MSKCC: Memorial Sloan Kettering Cancer Center. RARP: robot-assisted radical prostatectomy.

**Table 2:** Evidence from worldwide clinical guidelines on the utility of PSMA-PET.

Document Led by	Arguments for Using PSMA-PET	Arguments Against Using PSMA-PET
EAU [10]	PSMA-PET increases detection rates with respect to CT and BS, especially in high-risk PCa.	It is unclear whether patients with metastases detectable only with PSMA-PET should be managed using systemic therapies only, or whether they should be subjected to aggressive local and metastases-directed therapies. The prognosis and management of patients diagnosed as metastatic by this arm is unknown.
ESMO [11]	PSMA-PET has better sensitivity and specificity than CT or BS	PSMA-PET has not shown to improve clinical outcomes. Patients with localized disease on routine imaging should not be denied radical local treatment solely because metastatic lesions are identified on PSMA-PET. The evidence regarding PSMA-PET is not adequate to support recommendation concerning its use.
ASCO [12]	PSMA-PET is recommended if conventional imaging modalities are negative or equivocal in high- or very high-risk prostate cancer.	PSMA-PET is a costly test. Its high sensitivity for detecting low-burden disease may lead to incorrect patient management.
AUA [13]	Further investigations may establish the value of this test, but it would be recommended only for high-risk PCa patients.	PSMA-PET is an expensive test that is not recommended in the initial stage of PCa.
APCCC [14]	PSMA-PET should be used in high-risk localized PCa, but not in favorable intermediate-risk disease. The use of PSMA-PET in unfavorable intermediate-risk patients is controversial.	There was no consensus on how to treat patients who are M0 on conventional imaging but have positive lesions on PSMA-PET. Therapeutic decisions should be made with caution. Although it is possible that the use of PSMA-PET for staging may improve clinical outcomes by optimizing the use of local and/or adjuvant systemic therapy, this has yet to be proven.

EAU: European Association of Urology. PSMA-PET: prostate-specific membrane antigen positron emission tomography. CT: computed tomography. BS: bone scan. ESMO: European Society for Medical Oncology. ASCO: American Society of Clinical Oncology. AUA: American Urological Association. PCa: prostate cancer. APCC: Advanced Prostate Cancer Consensus Conference.

## 4. Discussion

The role of PSMA-PET in the staging and management of PCa is rapidly evolving. The advent of molecular imaging has reshaped the landscape of its diagnosis, particularly in high-risk patients. The superior sensitivity and specificity of PSMA-PET compared to conventional imaging techniques have prompted its inclusion in primary staging protocols for high-risk PCa, as recommended by the latest EAU guidelines.

An accurate evaluation of tumor extent at the beginning of the diagnosis is crucial for establishing the correct therapeutic strategy. The Tumour, Node, Metastasis (TNM) system of the American Joint Committee on Cancer and Union Internationale Contre le Cancer is the most used PCa staging system [22], along with the EAU risk group classification [23].

While CT, MRI and BS have traditionally been used for staging in patients with local, intermediate- to high-risk PCa, these modalities have limited precision in detecting small retroperitoneal lymph node metastases and small-volume bone metastases [8].

The likelihood of patients having positive LNs can be estimated using validated nomograms. As previously discussed, the Briganti [17] and MSKCC [18] nomograms are the most commonly utilized, both relying on similar clinical, biochemical and pathological preoperative variables. In contrast, the updated Briganti 201.9 [19] nomogram incorporates imaging findings and histological data from targeted biopsies following multiparametric MRI (mpMRI). Bilateral ePLND during RP is typically performed in cases where the risk of lymph node metastases exceeds 5%, and ePLND is considered the most accurate method for detecting LN involvement in PCa patients [8,24]. Unfortunately, we know it is an invasive diagnostic intervention associated with substantial complications. For these reasons, there is a need for more reliable imaging techniques for lymph node (LN) staging, and PSMA-PET has been thoroughly studied for its effectiveness in evaluating nodal staging [16].

A systematic review has recently been published by Dondi et al. They reported that this imaging modality may have the ability to correctly reclassify subjects according to their risk, guiding the choice of active surveillance (AS) and guiding the performance of biopsies for the investigation of high-grade CaP, thus avoiding AS [25].

PSMA is a cell-surface glycoprotein that is overexpressed in PCa cells. Radiolabeled small molecules that bind specifically to PSMA facilitate whole-body tumor imaging using PET-CT [6]. Two PSMA-targeting PET radiopharmaceuticals, ^68^Ga-PSMA-11 and 18F-DCFPyL, have gained U.S. Food and Drug Administration approval [26]. In addition, 18F-rhPSMA-7.3, a high-affinity PSMA-PET radiopharmaceutical, is in development as a diagnostic imaging agent for PCa [27]. 18F-PSMA-1007 has a similar biodistribution but reduced urinary clearance, enabling excellent assessment of the prostate. It demonstrated comparable results for PCa staging, with nearly perfect agreement in PCa lesion detection among the different tracers. Additionally, it exhibited an overall higher uptake in PCa lesions (measured as SUVmax) compared to other PSMA-targeted agents [28].

Maurer et al. [8] were the first to publish their results comparing ^68^Ga-PSMA-PET with CT and MRI in 130 patients with intermediate- to high-risk PCa scheduled for RP and ePLND. They concluded that preoperative nod staging with ^68^Ga-PSMA-PET demonstrated to be superior to standard imaging in these patients.

Hofman MS et al. published the ProPSMA study in 2020 [6], in which the investigators aimed to assess whether PSMA PET-CT had improved diagnostic accuracy when compared with the combination of CT and BS. The results suggested that in patients with high-risk PCa undergoing staging before curative-intent treatment, PET-PSMA should substitute conventional imaging modalities. However, the data provided by PSMA-PET and its subsequent management effects are unclear.

Van Kalmthout et al. [16] evaluated the diagnostic accuracy of ^68^Ga-PSMA-PET in the initial staging of PCa, assessing patients undergoing lymphadenectomy with ^68^Ga-PSMA-PET and reevaluating them after the test. They described a patient-based sensitivity of 41.5% (95% CI 26.7–57.8) for detecting LN metastasis, a patient-based specificity rate of 90.9% (95% CI 79.3–96.6) and positive and negative predictive values of 77.3% (95% CI 54.2–91.3) and 67.6% (95% CI 55.6–77.7), respectively. These findings resulted in a change in treatment in 13 patients (12.6%) based on the use of PSMA-PET. The clinical utility of PSMA-PET extends beyond mere detection. By integrating PSMA-PET findings into predictive nomograms, clinicians can more accurately assess the risk of LN metastases and tailor treatment strategies accordingly. This is particularly relevant in guiding the decision to perform ePLND, an invasive procedure with significant morbidity.

In a multicenter, international study involving patients who underwent robot-assisted RP and ePLND, the effectiveness of three established preoperative nomogram models [17,18,19] was evaluated for predicting pN1 disease. The study also assessed whether incorporating PSMA-PET imaging could enhance the predictive accuracy of these models [21]. The findings indicated that adding PSMA-PET to the existing nomograms significantly improved their ability to accurately predict the outcomes of ePLND.

Despite these advances, caution is recommended in interpreting PSMA-PET findings, especially when it comes to indicate a change in therapeutic management [4,6,29]. The absence of prospective studies demonstrating a survival benefit from PSMA-PET-driven interventions underscores the need for a measured approach. The EAU guidelines emphasize that while PSMA-PET can enhance diagnostic accuracy, its impact on long-term outcomes remains to be definitively proven.

A recent study evaluated the usefulness of PSMA-PET in deciding whether to perform an ePLND [24], highlighting the consequent increase in unnecessary procedures. Another study was designed to identify patients at higher risk of advanced-stage disease [30]. The first one emphasizes that the tools for predicting LN metastases are associated with suboptimal performance for men with N0M0 PCa. The latter concluded that patients with ISUP (International Society of Urological Pathology) grade 2–3, as well as patients with organ-confined disease at mpMRI and a single or two positive nodal findings at PET are those in whom RP may achieve the best oncological effects in the context of a multimodal approach.

Summarizing the recommendations of existing clinical guidelines, EAU guidelines [10] reference several multicentric studies demonstrating that PSMA-PET increased detection rates compared to conventional imaging modalities due to its sensitivity and specificity, especially in high-risk PCa patients [6,10,31]. However, in the absence of prospective studies demonstrating survival benefits, caution must be exercised when making therapeutic decisions [10,32]. The ESMO guidelines [11] confirm that men with intermediate- or high-risk disease should undergo imaging for nodal or metastatic disease, with PSMA-PET offering better sensitivity and specificity than CT or BS [10,31]. However, it has not been shown to improve clinical outcomes. For these reasons, they argue that patients with localized disease on routine imaging should not be denied radical local treatment just because metastatic lesions are identified on novel imaging techniques [11]. The ASCO guidelines [12] state that PSMA-PET shows an excellent sensitivity, but it also has several disadvantages, especially because it is costly, and its high sensitivity for detecting low-burden disease may lead to incorrect patient management in some cases. They concluded that PET-PSMA is recommended if conventional imaging modalities are negative or equivocal in high- or very high-risk prostate cancer. The AUA guidelines [13] show that PSMA-PET is not recommended in the initial stage of PCa. The APCCC held in 2022 [14] states that PSMA-PET should be requested in cases of high-risk localized PCa. It should not be used in favorable intermediate-risk disease, given that its use remains controversial in unfavorable intermediate-risk patients. However, the APCCC agrees with incorporating the results of PSMA-PET into a new classification of TNM. On the other hand, there was no agreement on how to treat patients who are M0 on conventional imaging but have positive lesions on PSMA PET [15,33].

### Why Should We Limit the Use of PSMA in Primary Staging?

PSMA PET/CT is still characterized by limited sensitivity and, at present, cannot replace an ePLND. According to Jansen at al., in their prospective cohort study involving 117 patients, it demonstrated high specificity (94.4%) but limited sensitivity (41.2%) for the detection of PLN metastases in primary PCa [34]. Similar results were reported in a prospective multicenter phase II/III study, which showed a mean specificity of 97.9% (95% CI 94.5–99.4%) and a mean sensitivity of 40.3% (28.1–52.5%) for detecting pelvic lymph node involvement [35]. This suggests that PSMA-based PET/CT cannot yet replace ePLND.

The phase 3 LIGHTHOUSE study [36] investigated the use of 18F-rhPSMA-7.3 in men with newly diagnosed PCa, who were scheduled for robot-assisted RP with ePLND. This study is notable for being one of the few in which all PSMA PET/CT images were analyzed blindly by three independent readers, with histopathological analysis of LND specimens serving as the reference standard. The sensitivity of 18F-rhPSMA-7.3 PET/CT for detecting lymph node metastasis was found to be low, ranging from 23% to 30% among the three readers, but increased to 38% and to 52% for ISUP grade 5 cancer, likely due to higher PSMA expression in higher-grade tumors [37]. This low sensitivity aligns with findings from the OSPREY [38] and UCLA/UCSF [39] prospective multicenter trials, which reported sensitivities ranging from 30% to 40% for 18F-DCFPyL and ^68^Ga-PSMA-11 in patients with negative or equivocal standard imaging who underwent LND. However, the reported sensitivity of PSMA PET/CT in these studies may be underestimated, as it does not account for patients who did not undergo LND, primarily due to metastatic disease identified after PSMA imaging [36]. Consequently, it is anticipated that guideline recommendations will shift towards first-line PSMA PET/CT staging for high-risk and very high-risk cancers, leading clinicians to expect higher sensitivity from PSMA imaging than has been reported.

Currently, prospective studies demonstrating a survival benefit are lacking, so caution is advised when making therapeutic decisions [40]. Therefore, it is time to move away from using PSMA imaging as a standalone dual modality and focus our research efforts on integrating PSMA imaging findings with other clinicopathological data to enhance clinical outcomes [41].

PSMA-PET is a powerful tool in the diagnostic armamentarium for prostate cancer, particularly in high-risk cases. Its ability to detect metastases with greater accuracy than conventional imaging has the potential to change the course of treatment in a significant subset of patients. However, the integration of PSMA-PET into clinical practice should be accompanied by a thorough understanding of its limitations and the ongoing need for evidence-based decision-making.

Our work reviews the evidence described so far on the use of this novel technique in the estimation of localized prostate cancer, especially in patients with intermediate- and high-risk disease. However, the low number of prospective comparative studies and randomized clinical trials, as well as the low number of systematic reviews and meta-analyses, is a limitation that we aim to address in the coming years, as more scientific evidence becomes available.

## 5. Conclusions

In the era of next-generation imaging, PSMA-PET has emerged as a pivotal technology for the initial staging of high-risk prostate cancer. Its enhanced sensitivity and specificity over conventional imaging modalities offer a more precise evaluation of disease extent, particularly in detecting nodal and distant metastases. Nevertheless, the absence of definitive evidence linking PSMA-PET findings to improved survival outcomes necessitates a cautious approach in clinical decision-making. As the landscape of prostate cancer management continues to evolve, further prospective studies are essential to fully elucidate the role of PSMA-PET in improving patient outcomes.

## Data Availability

Not applicable.
